# Carbofuran accelerates the cellular senescence and declines the life span of *spns1* mutant zebrafish

**DOI:** 10.1111/jcmm.16171

**Published:** 2020-12-04

**Authors:** Alam Khan, Talukdar Mohammad Fahad, Tanjima Akther, Tanjeena Zaman, Md Faruk Hasan, Md Rafiqual Islam Khan, Mohammad Saiful Islam, Shuji Kishi

**Affiliations:** ^1^ Department of Pharmacy University of Rajshahi Rajshahi Bangladesh; ^2^ Department of Molecular Medicine The Scripps Research Institute Jupiter FL USA; ^3^ Department of Fisheries University of Rajshahi Rajshahi Bangladesh; ^4^ Department of Biology University of Hail Hail Saudi Arabia; ^5^ Department of Genetic Engineering and Biotechnology University of Rajshahi Rajshahi Bangladesh; ^6^ S&J Kishi Research Corporation Jupiter FL USA

**Keywords:** carbofuran, life span, senescence, spinster homolog 1, zebrafish

## Abstract

Carbofuran is a carbamate pesticide, widely used in agricultural practices to increase crop productivity. In mammals, carbofuran is known to cause several untoward effects, such as apoptosis in the hippocampal neuron, oxidative stress, loss of memory and chromosomal anomalies. Most of these effects are implicated with cellular senescence. Therefore, the present study aimed to determine the effect of carbofuran on cellular senescence and biological ageing. Spinster homolog 1 (Spns1) is a transmembrane transporter, regulates autolysosomal biogenesis and plays a role in cellular senescence and survival. Using senescence‐associated β‐galactosidase staining, we found that carbofuran accelerates the cellular senescence in *spns1* mutant zebrafish. The yolk opaqueness, a premature ageing phenotype in zebrafish embryos, was accelerated by carbofuran treatment. In the survival study, carbofuran shortened the life span of *spns1* mutant zebrafish. Autophagy is the cellular lysosomal degradation, usually up‐regulated in the senescent cells. To know the impact of carbofuran exposure on autophagy progress, we established a double‐transgenic zebrafish line, harbouring EGFP‐tagged LC3‐II and mCherry‐tagged Lamp1 on *spns1* mutant background, whereas we found, carbofuran exposure synergistically accelerates autolysosome formation with insufficient lysosome‐mediated degradation. Our data collectively suggest that carbofuran exposure synergistically accelerates the cellular senescence and affects biological ageing in *spns1* defective animals.

## 1 INTRODUCTION

Senescence is a cellular process that implements permanent cell cycle arrest in response to various stimuli, such as oxidative stress, telomere loss, chemotherapeutic drugs, DNA damage and oncogenic signalling.^1^, ^2^ The elevated intensity of senescent cells mainly exists in ageing tissues, because of its implication with ageing.^1^ Ablation of senescent cells by mechanical or other means improve health outcome as well as prolongs biological ageing.^3^ Activation of senescence‐associated beta‐galactosidase (SA‐β‐gal) in the senescent cell is considered as the hallmark of cellular senescence.^4^, ^5^ Although the maximum beta‐galactosidase activity can be seen at medium to strong acidic pH, the least activity that can be found at pH 6, which typically uses to examine the senescent and ageing cells.^6^ On the other hand, autophagy is a cellular lysosomal degradation process, essential to regulate energy homeostasis through recycling unnecessary or dysfunctional cytoplasmic constituents.^7^ In normal cells, autophagy occurs at low baseline altitudes, which is elevated in the senescent cells.^8^ Autophagy is initiated with the formation of the phagophore from a variety of sources, such as ER‐mitochondrial junctions, ER‐plasma membrane contact sites and ER‐Golgi intermediate compartment. Then, phagophore wraps unnecessary or dysfunctional cytoplasmic constituents to build‐up autophagosomes, which successively fuse with lysosomes to form autolysosomes.^9^, ^10^ Finally, through the utilization of lysosomal hydrolases, the contents of autophagosomes are degraded and recycled. The vacuolar H^+^‐ATPase (V‐ATPase) is a proton pump, which establishes and sustains an acidic condition within the lysosome. The V‐ATPase promotes autophagosome‐lysosome fusion and lysosome‐mediated degradation of the contents of autophagosomes.^11^

Lysosomal degradation yields are released into the cytosol through the lysosomal efflux transporter system. Spinster homolog 1 (Spns1) in vertebrates is known as Spinster (Spin) in flies, encodes a putative lysosomal efflux permease, whose amino acid sequence has resembled the amino acid sequence of lysosomal sugar carrier.^12^ The spin mutation is associated with neurodegeneration, rejection behaviour against the opposite sex and shortened life span in flies.^13^ In zebrafish, the loss of *spns1* gene leads to the accumulation of opaque substances in the yolk, and yolk extension and reduces biological ageing.^12^, ^14^ Recently, we found that the concurrent disruption of the V‐ATPase subunit gene, *ATPase, H^+^ transporting, lysosomal, V0 subunit Ca* (*atp6v0ca*) in *spns1* mutant fish synergistically induced cellular senescence and shorten the life span.^15^

Carbofuran is a toxic carbamate pesticide, whose chemical name is 2,3‐dihydro‐2,2‐dimethyl‐7‐benzofuranyl methylcarbamate.^16^ It is widely used to control pests in agricultural fields, particularly in developing countries.^17^, ^18^ It has insecticidal, nematicidal and acaricidal activities.^19^, ^20^ In Bangladesh, 37% of the total sold pesticides during 2007 was carbofuran.^21^ Humans, particularly in agricultural dependent countries, are often exposed to pesticides including carbofuran through contaminated water, air and vegetables.^22^, ^23^ The World Health Organization (WHO) classified carbofuran as a highly hazardous pesticide (category 1b).^24^ Its toxicity to mammals leads to sperm abnormalities and chromosomal aberrations.^23^ Carbofuran suppresses anti‐oxidative enzymes in the body, such as glutathione‐*S*‐transferase and superoxide dismutase, and induces oxidative stress through the generation of reactive oxygen species (ROS).^25^, ^26^ It causes apoptosis of hippocampal neurons through inducing DNA fragmentation,^27^ also causes Alzheimer's syndrome type pathology in the central nervous system.^28^ Furthermore, carbofuran reduces ATP generation in neurons through the inhibition of glycolysis and Kreb's cycle.^29^, ^30^

The detrimental effects of carbofuran on the anti‐oxidative defence system as well as on the nervous system suggest its implication in cellular senescence and biological ageing. However, the role of carbofuran on cellular senescence and/or biological ageing still remains obscure. In the present study, we found that carbofuran synergistically accelerates the cellular senescence and shortens the life span of *spns1* mutant zebrafish. In addition, using a transgenic zebrafish line [Tg(EGFP‐LC3); spns1^+/−^], expressing EGFP‐tagged microtubule‐associated protein 1 light chain 3‐II (LC3‐II) on autophagosome, it was revealed that carbofuran affects the autophagy process in *spns1* mutant fish.

## 2 MATERIALS AND METHODS

### 2.1 Chemicals and reagents

Carbofuran of analytical grade was procured from Merck, Germany (SKU 32056). Its stock solution was prepared by solubilizing in DMSO, which was diluted in egg water to prepare the final solution for treatment. The solution to be used for the control group was prepared likely by diluting an equal volume of DMSO in egg water. Reagents such as potassium ferrocyanide, potassium ferricyanide, 1‐phenyl‐2‐thiourea, paraformaldehyde and MgCl_2_ were purchased from Sigma‐Aldrich, Germany (SKU P3289, 702587, P7629, 16005 and M8266).

### 2.2 Zebrafish husbandry

Adult zebrafish of both wild and mutant types were harboured under a 14‐hour light/10‐hour dark cycle at 25°C. Zebrafish embryos were maintained and raised at 28.5°C under the same light‐dark cycle. After three weeks of embryonic development, all embryos were maintained under adult fish condition. Zebrafish were fed with both brine shrimp and flake food, each once daily. Using a circulation system, water of zebrafish tanks were replaced at each 10‐15 minutes interval. Hours of post‐fertilization (hpf) of Kimmel et al^31^ was considered to determine the developmental stages of embryos.

### 2.3 Generation of transgenic zebrafish

PT2‐EGFP‐LC3 and PT2‐mCherry‐Lamp1 plasmids were kindly donated by Dr Shuji Kishi Lab of the Scripps Research Institute, Florida, USA. Tol2 transposase mRNA was synthesized using SP6 RNA polymerase kit (Ambion, AM1340). Collected plasmids concurrently with Tol2 transposase mRNA were microinjected into embryos of the one‐cell stage. Embryos having significant green and/or red fluorescent expression under the fluorescence condition of the microscope were sorted, raised to adulthood and mated with wild fishes to find germline‐transmitted embryos. Embryos having a green or red fluorescent expression of nearly similar intensity were founder zebrafish (F0) embryos, which were raised to adulthood and incrossed to give F1 generation as described.^32^ Adult F1 fishes were mated, and resulting embryos were used in experiments.

### 2.4 Senescence‐associated β‐galactosidase activity assay

Embryonic senescence caused by chemical‐induced oxidative stress and gene mutation can be detected using the SA‐β‐gal assay.^33^ A chemical reagent, 5‐bromo‐4‐chloro‐3‐indolyl β‐d‐galactopyranoside, is widely used for SA‐β‐galactosidase staining, and their interactions are signposted by blue staining. Embryos were washed with phosphate buffer saline (PBS) solution and fixed in 4% paraformaldehyde. After around 12 hours of fixation, embryos were washed three times with PBS solution and two times with staining buffer. The staining buffer was prepared by dissolving potassium ferricyanide (5 mmol/L), potassium ferrocyanide (5 mmol/L) and MgCl_2_ (2 mmol/L) in PBS solution, where the pH of the solution was adjusted around 6.0 using NaOH and/HCl. Staining was conducted by immersing embryos in the staining buffer, which containing 5‐bromo‐4‐chloro‐3‐indolyl β‐d‐galactopyranoside (X‐gal) at the concentration of 20 μg/ml. The embryonic preparation was kept at 10°C until sufficient staining. Then, embryos were re‐washed four times with PBS solution and photographed. The staining intensity of the captured images was quantified using Adobe Photoshop CS software.^14^

### 2.5 Chemical treatments

Zebrafish embryos were treated at a number of late developmental stages. All treatments were performed with a six‐well plate. Based on the literature survey, several concentrations of carbofuran (10, 100 and 200 μmol/L) were prepared to select the maximum effective concentration (without inducing toxic morphological abnormality). The maximum effect (SA‐β‐gal activity) was found at 100 μmol/L concentration of carbofuran (Figure 2B), and the concentration did not significantly affect the morphological phenotype of embryos. The higher concentration of carbofuran (200 μmol/L) was excluded from subsequent experiments because of its shortening effect on the body length of zebrafish embryos (data have not shown). The applied carbofuran solution was replaced by a fresh carbofuran solution at each 12 hours interval. Treated embryos were maintained at 28.5°C, under 14‐hour light/10‐hour dark cycle. During treatment, embryos were observed under a microscope at each 3 hours interval. All abnormalities or deaths observed were recorded, and dead embryos were removed from the treatment solution. All experiments were approved by the Institutional Animal, Medical Ethics, Biosafety, and Biosecurity Committee of the Institute of Biological Science (I. B. Sc.) of the University of Rajshahi, Bangladesh (245/451/IAMEBBC/IBSc). The guidelines of I. B. Sc. were followed in animal handling.

### 2.6 Heterozygote *spns1^+^* ^/^ *^hi891^* fish identification

Heterozygote *spns1^+^* ^/^ *^hi891^* (hereafter *spns1^+^* ^/−^) fish was kindly donated by Dr Shuji Kishi Lab of the Scripps Research Institute, Florida, USA. To identify heterozygote *spns1^+^* ^/−^ fishes, DNA was extracted from the tail fin of zebrafish, and then, genomic and mutant sequences of heterozygote *spns1^+^* ^/−^ fishes were amplified using PCR reactions. The primers used are *spns1* genomic forward (5′‐AGGTAAAGACAGCCCGAAAC‐3′), *spns1* genomic reverse (5′‐GATCCCAGACGCCAACATTA‐3′), *spns1* (*hi891*) mutant forward (5′‐ TAAGTCGGTCGGCTGCACGGTT‐3′), and *spns1* (*hi891*) mutant reverse (5′‐ TGATCTCGAGTTCCTTGGGAGGGTCT −3′). Wild fish (*spns1^+^* ^/+^) harbouring only *spns1* genomic sequence, whereas heterozygote fish (*spns1^+^* ^/−^) harbouring both *spns1* genomic and *spns1* (*hi891*) mutant sequences, and homozygote *spns1^hi891^* ^/^ *^hi891^* fish (hereafter *spns1* ^−/−^) has only *spns1* (*hi891*) mutant sequence.^34^

### 2.7 Lysosomal staining

At the beginning of the autophagy process, microtubule‐associated protein 1 light chain 3‐II (LC3II, hereafter mentioned as LC3) is formed at the inner membrane of the autophagosome.^15^, ^35^ Transgenic zebrafish expressing EGFP‐LC3 on *spns1^+^* ^/−^ heterozygote background [Tg(EGFP‐LC3); spns1^+/−^] was used to estimate the progress in autophagy. Although autophagy keeps in progress, autophagosomes are fuses with the lysosomes to form autolysosomes, where the content of autophagosomes is sequestered by the lysosomal enzymes.^32^, ^36^ To estimate the co‐localization of the autophagosome with the lysosome, LysoTracker Red DND‐99 (Invitrogen/Molecular Probes, L7528) was used to stain the lysosome. Staining was carried out by incubating embryos at 28.5°C for 1 hour in PBS solution, which containing LysoTracker Red DND‐99 at the concentration of 0.005 μmol/L.

### 2.8 Microscopy and imaging

Imaging was carried out using both macrofluorescence microscope (Nikon AZ100) and confocal microscope (Olympus; FluoView 1000). Before live imaging, embryos were anaesthetized with tricaine solution (0.16 mg/ml). The whole body of SA‐β‐gal stained fish was photographed using the reflected bright light of the Nikon AZ100 microscope. In addition, the cellular SA‐β‐gal staining signal was observed under the confocal microscope. In each experiment, all images were taken under the same condition of the microscope.

### 2.9 RT‐PCR analysis

Regulations of mRNA levels of *pai1*, *p21* and *smp30* genes were estimated by RT‐PCR analysis. Total RNA was isolated using TRIzol reagent (Invitrogen, 15596026), and double‐strand cDNA was synthesized using M‐MLV reverse transcriptase (Promega, M1705). RT‐PCR primers sequences were presented in Table 1 including their annealing temperatures and amplification cycle.

**TABLE 1:** List of primers used for RT‐PCR analysis

Genes	Primers	Annealing temp (°C)	Cycles
*pai‐1*	Forward: CTGATCTTTGCCCTTTGCGCATCA Reverse: TTTGCTCAAGCTGCGCCTAAAGAC	55	25
*p21*	Forward: TGAGAACTTACTGGCAGCTTCA Reverse: ACGTGCATTCGTCTCGTAGC	55	25
*smp‐30*	Forward: ACTATGACATCCAAACTGGAGGA Reverse: CTTCTGTGTCTATGCACATACCG	51	25
*β‐actin*	Forward: CCCAGACATCAGGGAGTGAT Reverse: CACCGATCCAGACGGAGTAT	60	18

Note

During PCR amplification, the initial denaturing was carried out at 94°C for 5 min. Amplicon size (bp) of RT‐PCR products were 354, 396, 98 and 896 for *pai‐1*, *p21*, *smp30* and β‐*actin*, respectively.

### 2.10 Quantitative analysis

Intensities of fluorescence of captured images were quantified using Adobe Photoshop CS software. PCR bands were quantified by ImageJ software (Java‐based image processing program developed by NIH). Data analysis was carried out using SPSS statistics software (version 15.0). Data are presented as mean ± SEM. A comparison among different groups was made by Student's *t* test.

## 3 RESULTS

### 3.1 Carbofuran synergistically accelerates the senescence‐associated‐β‐galactosidase activity in *spns1* ^−/−^ mutant zebrafish

**FIGURE 1:**
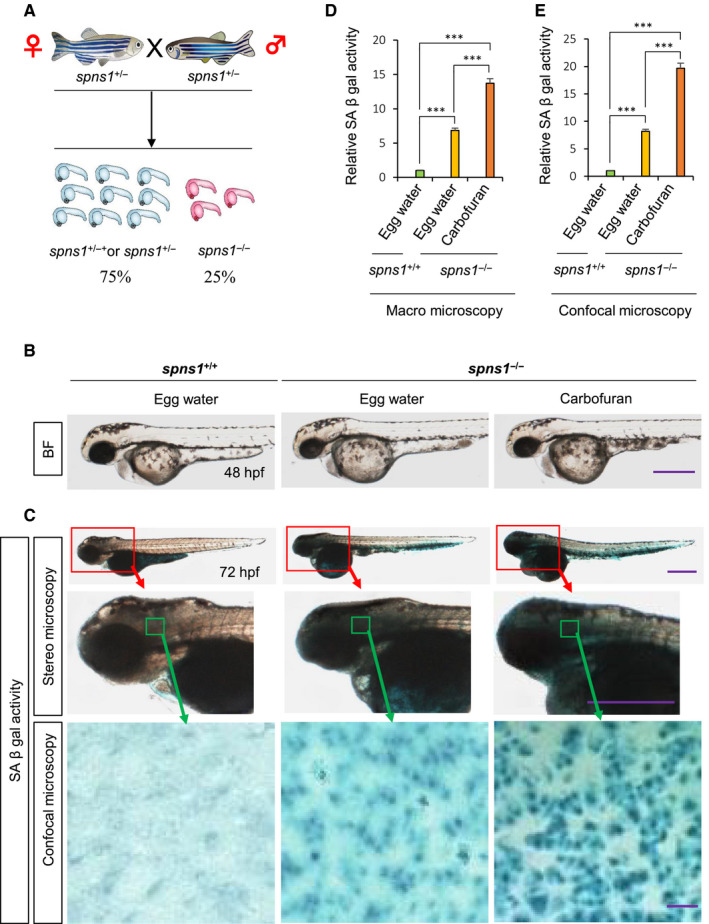
Carbofuran accelerated yolk opaqueness and SA‐β‐galactosidase activity in *spns1* mutant zebrafish. A, Incross among heterozygote (*spns1* ^+/−^) fishes. Around 25% of resultant embryos are *spns1* ^−/−^ homozygous mutant, whereas the remaining 75% of embryos consist of wild (*spns1* ^+/+^) and heterozygous (*spns1* ^+/−^) embryos. B, Yolk opaqueness phenotype in homozygote (*spns1* ^−/−^) fish. The opaqueness was accelerated by carbofuran exposure. C, The SA‐β‐gal activity was high in homozygote (*spns1* ^−/−^) fish and accelerated by exposure to carbofuran. The SA‐β‐gal staining at the head was expanded, as shown by a red box and red arrow. The SA‐β‐gal staining of whole fish was observed under the bright field (BF) condition of the stereo‐microscope. The cellular SA‐β‐gal staining in the head (shown by green box and arrow) was observed by confocal microscopy. The scale bars are 250 mm (stereo microscopic images) and 10 mm (confocal microscopic images). D, Quantification of SA‐β‐gal staining intensity of whole fish. E, Quantification of cellular SA‐β‐gal staining signals in the head region. The number of animals was 10 (n = 10). ***P ≤ .005

When heterozygote *spns1^+^* ^/−^ adult fishes were incrossed, around 25% of resultant embryos have shown homozygous (*spns1* ^−/−^) phenotypes (Figure 1A). The most prominent morphological phenotype in homozygous embryos was yolk opaqueness. Although homozygous fishes had a short life span (4‐6 days), heterozygous fishes developed into adulthood normally without any yolk opaqueness phenotype.^14^, ^34^ Increased SA‐β‐gal activity because of the senescence induction was established in *spns1* ^−/−^ mutant fish.^12^, ^14^ We confirmed both the yolk opaqueness phenotype and the up‐regulation of SA‐β‐gal activity in our homozygous fishes (Figure 1B,C). Besides, a much stronger yolk opaqueness phenotype was found in carbofuran treated homozygous (*spns1* ^−/−^) fish (Figure 1B). The SA‐β‐gal staining intensity throughout the whole body of fish was examined under a stereo‐microscope (macromicroscopy). In addition, blue particles of SA‐β‐gal staining at the cellular level around the head region (dorsal to the eye) were determined by a confocal microscopic examination. Carbofuran treatment accelerated SA‐β‐gal activity under both microscopic observations (Figure 1D,E). Macromicroscopic observation particularly indicated an enhancement of SA‐β‐gal staining intensity at the head and tail regions (Figure 1C), whereas the carbofuran treated group showed the highest intensity compared to other groups. These observations suggest that carbofuran synergistically accelerated senescence activity in *spns1* ^−/−^ mutant fish.

### 3.2 Carbofuran exacerbates premature ageing phenotype and lifespan in *spns1* ^−/−^ mutant zebrafish

**FIGURE 2:**
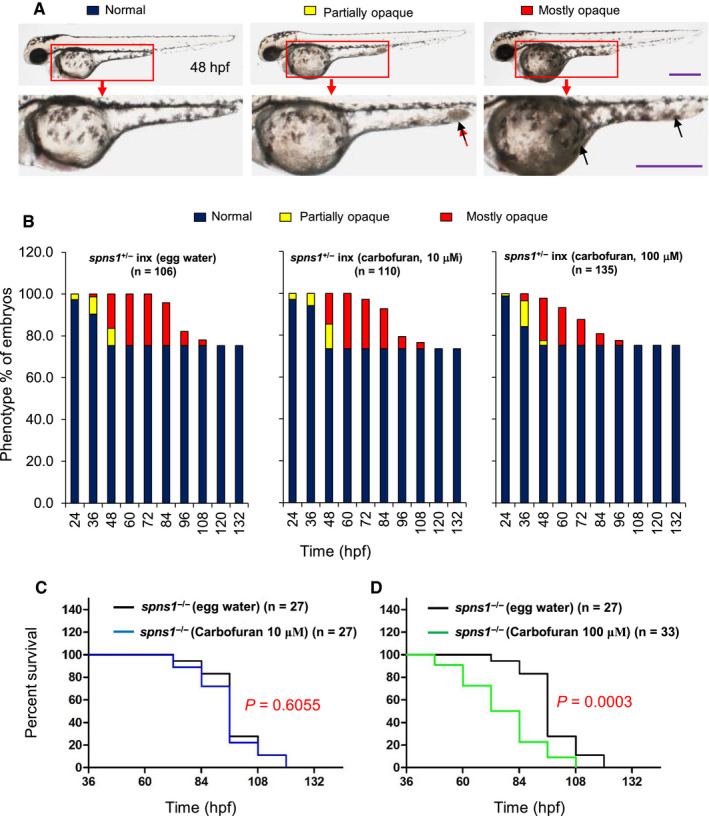
Carbofuran exacerbated the *spns1* deficiency phenotype and shortened the survival of *spns1* mutant zebrafish. A, Sorts of yolk opaqueness phenotypes in *spsn1* ^−/−^ (homozygous) zebrafish based on the extent of opacity in the yolk and/or yolk extension: partially opaque (yellow colour) and mostly opaque (red colour). Wild (*spns1* ^+/+^) and heterozygous (*spns1* ^+/−^) zebrafish do not show such opaqueness (dark blue colour). B, Effect of carbofuran on the opaqueness phenotype of *spns1* mutant fish and death record. The opaqueness phenotype and death records were not affected by 10 μmol/L carbofuran exposure. However, the mostly opaque phenotype and death of embryos of 100 μmol/L carbofuran treated group were found at earlier times than fishes of the control group (egg water treatment). C, Survival curves of *spns1* mutant fishes for carbofuran treatment at 10 and 100 μmol/L concentrations. By 100 μmol/L carbofuran exposure, the survival of *spsn1* mutant fishes was significantly declined (*P* = .0003)

Embryonic senescence has been implicated in the regulation of the ageing process. Accelerated senescence can trigger ageing symptoms and declines organismal longevity.^5^, ^14^ A premature ageing phenotype in *spns1* ^−/−^ mutant zebrafish is yolk opaqueness, which typically begins from the posterior end of yolk extension and gradually progresses towards other parts of yolk extension and yolk.^14^, ^15^ If a small part of the yolk and/or yolk extension of the embryo becomes opaque, then it is denoted as a ‘partially opaque.’ On the other hand, when the major part of yolk and yolk extension become opaque, then it is denoted as ‘mostly opaque’ (Figure 2A).^15^ Mostly opaque embryos usually die within the subsequent 24‐48 hours.

Heterozygous *spns1* ^+/−^ adult fishes were incrossed, and resultant embryos were treated with low (10 μmol/L) and high (100 μmol/L) doses of carbofuran since 20 hpf. Treated embryos were monitored at each 12 hours intervals for yolk opaqueness phenotype and survival data. Among embryos of both control and carbofuran treated groups, around 25% have revealed yolk opaqueness phenotype (Figure 2B). Yolk opaqueness phenotype of embryos of low‐dose carbofuran treatment group was almost similar to that of the control group. Almost all embryos of high‐dose carbofuran treated group became mostly opaque at 48 hpf, whereas remarkable parts of embryos of both control and low‐dose carbofuran treated groups remained partially opaque (Figure 2B), suggesting that carbofuran treatment (100 μmol/L) exacerbated the premature ageing phenotype in *spns1* ^−/−^ mutant fish. In addition, among embryos of the high‐dose carbofuran treated group, mostly opaque embryos died at an earlier time than that observed in the case of embryos of other groups (Figure 2B). Kaplan‐Meier survival analysis for *spns1* ^−/−^ mutant larvae showed that the survival patterns of low‐dose carbofuran (10 μmol/L) treated larvae were nearly similar to the survival patterns of larvae of the control group, whereas the survival of larvae treated with 100 μmol/L of carbofuran was significantly shortened (Figure 2C).

### 3.3 Carbofuran accelerates autophagy formation in *spns1* ^−/−^ mutant zebrafish

**FIGURE 3:**
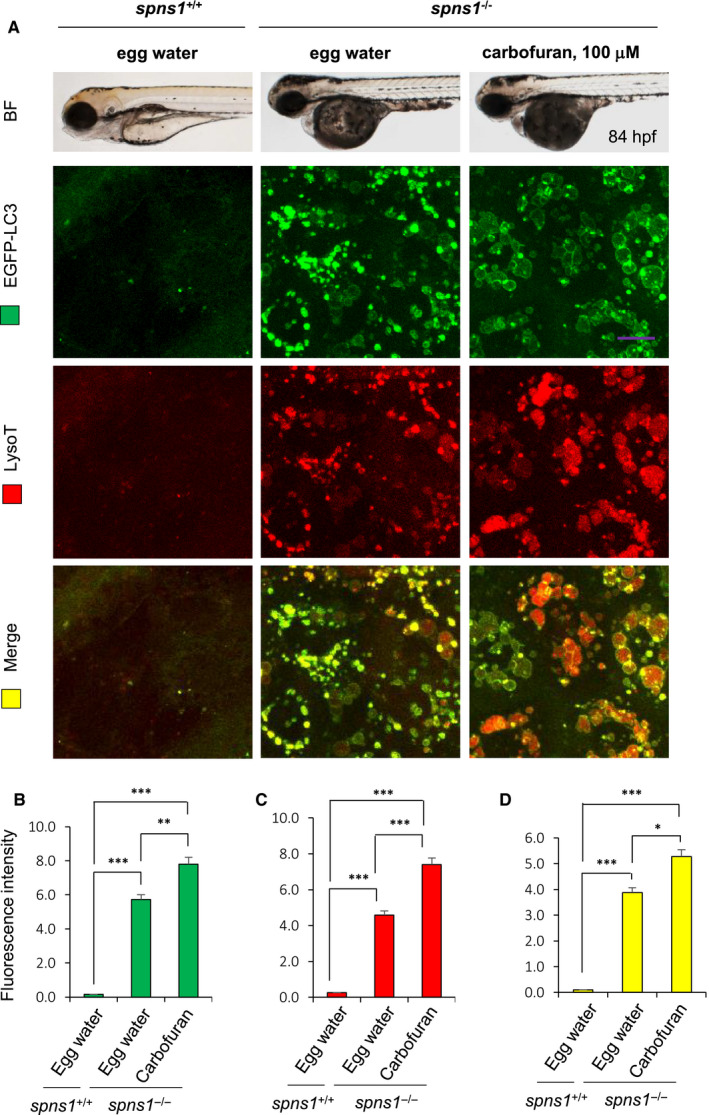
Carbofuran accelerated autolysosomal puncta formation in *spns1* mutant zebrafish. A, Carbofuran treatment worsened the yolk opaqueness phenotype of *spns1* mutant fishes, and most embryos lost their yolk extension. Loss of the *spns1* gene increased autophagosomal EGFP‐LC3 and lysosomal LysoTracker (LysoT) red expressions, which were further accelerated by carbofuran exposure. Most LysoTracker red expressions were co‐localized with EGFP‐LC3 expressions (bottom row; yellow colour). B, Quantification of autophagosomal EGFP‐LC3 expression. C, Quantification of LysoTracker red expression. D, Quantification of the merged expression (co‐localization). These expressions were significantly increased by the loss of *spns1* gene and were further significantly up‐regulated by carbofuran exposure. The scale bar is 10 mm; n = 6; *P ≤ .05; **P ≤ .01; ***P ≤ .005

Cellular autophagy is essential to maintain cellular homeostasis.^11^ It has an advantageous effect on organismal longevity.^12^ Under stress conditions of the body such as growth factor withdrawal, hypoxia, starvation, tumour progression and ageing, autophagy is up‐regulated above the normal baseline level.^9^ During autophagy, cytosolic microtubule‐associated protein 1 light chain 3‐II (LC3) associated autophagosomes are fuses to lysosomes and subsequently sequestered by lysosomal enzymes.^10^, ^12^ We have EGFP‐tagged LC3 transgenic zebrafish on *spns1* ^+/−^ (heterozygous) background. Mutant (*spns1* ^−/−^) embryos were treated with carbofuran since 40 hpf and subjected to lysosomal red staining before imaging. Excess autolysosome formation in *spns1* ^−/−^ mutant fishes above normal limit was suggested by Sasaki et al.^12^ Our current experiment reconfirmed his finding and additionally found carbofuran treatment synergistically accelerates autolysosome formation in *spns1* ^−/−^ mutant fish. Both autophagosomal (represented by EGFP‐tagged LC3) and lysosomal (represented by LysoTracker red) expressions were significantly increased in carbofuran treated *spns1* ^−/−^ mutant fish in comparison to the fishes of other groups (Figure 3A‐C). The co‐localization of autophagosomal and lysosomal expressions was also found significant (merge images, yellow colour; Figure 3A,D), suggesting autophagosomes were fused with lysosomes to form autolysosomes. However, because of insufficient lysosome‐mediated degradation, the autolysosomes were accumulated in cells.

**FIGURE 4:**
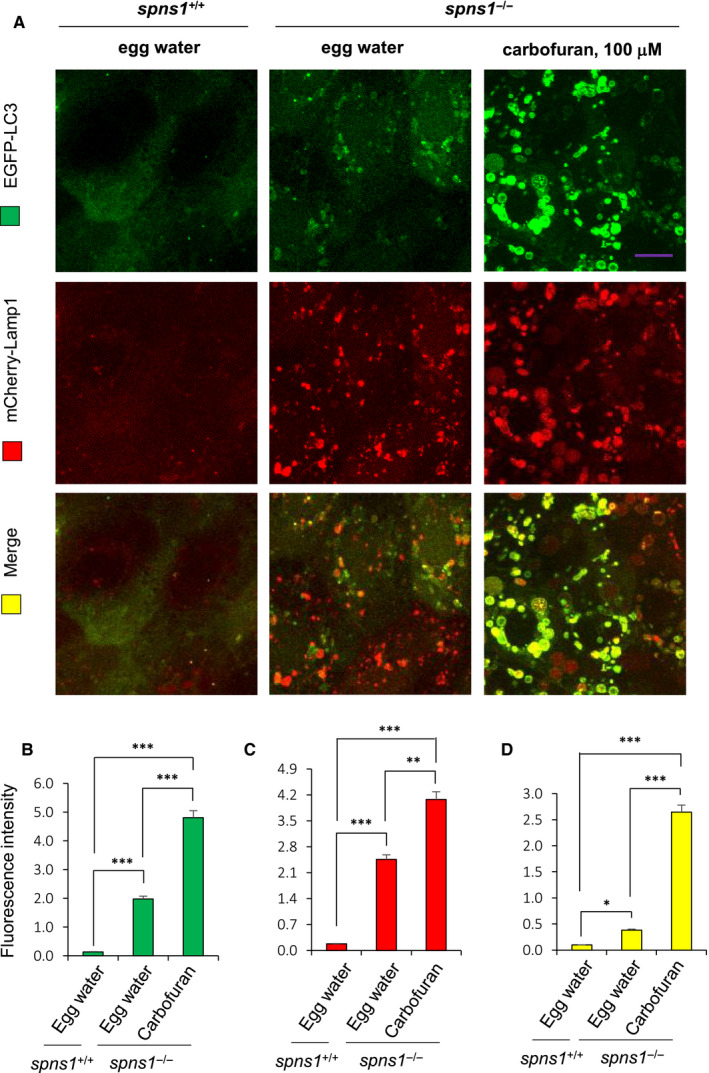
Carbofuran accelerated autolysosomal puncta formation in a double‐transgenic zebrafish line of *spns1* mutant background, expressing EGFP‐LC3 and mCherry‐Lamp1. A, Carbofuran exposure accelerated both autophagosomal EGFP‐LC3 and lysosomal mCherry‐Lamp1 expressions in *spns1* mutant zebrafish. Lysosomal mCherry‐Lamp1 expression was merged to EGFP‐LC3 expression (bottom row; yellow colour). B, Quantification of autophagosomal EGFP‐LC3 expression. C, Quantification of lysosomal mCherry‐Lamp1 expression. D, Quantification of the co‐localization of expressions. Both EGFP‐LC3 and mCherry‐Lamp1 expressions (with their co‐localization) were significantly increased by the loss of the *spns1* gene and were further significantly up‐regulated by carbofuran exposure. The scale bar is 10 mm; n = 6; *P ≤ .05; **P ≤ .01; ***P ≤ .005

The lysosomal‐associated membrane protein 1 (Lamp1) is a glycoprotein encoded by the *Lamp1* gene, widely used as a lysosomal membrane marker.^37^ To further confirm the co‐localization of autophagosomal expression to lysosomes, we used a double‐transgenic *spns1* ^+/−^ heterozygous fish, expressing EGFP‐tagged LC3 and mCherry‐tagged Lamp1. We found that both autophagosomal EGFP‐LC3 and lysosomal mCherry‐Lamp1 expressions were excessively aggregated in carbofuran treated *spns1* ^−/−^ mutant fish compared to the fishes of other groups (Figure 4A‐C). Co‐localization of EGFP‐LC3 vesicles to mCherry‐Lamp1 vesicles was more significant in carbofuran treated *spns1* ^−/−^ mutant fish than that found in the case of *spns1* ^−/−^ mutant fish (Figure 4A,D). Furthermore, in *spns1* ^−/−^ mutant fish, both autophagosomal and lysosomal vesicle sizes were increased and the increments were accelerated by the carbofuran treatment (Figures 3A and 4A). These results together suggest that the effect of carbofuran on the ageing of *spns1* ^−/−^ mutant fish has mediated through its detrimental effect on the cellular autophagic process.

### 3.4 Carbofuran affects ageing‐associated genes regulation in *spns1* ^−/−^ mutant zebrafish

**FIGURE 5:**
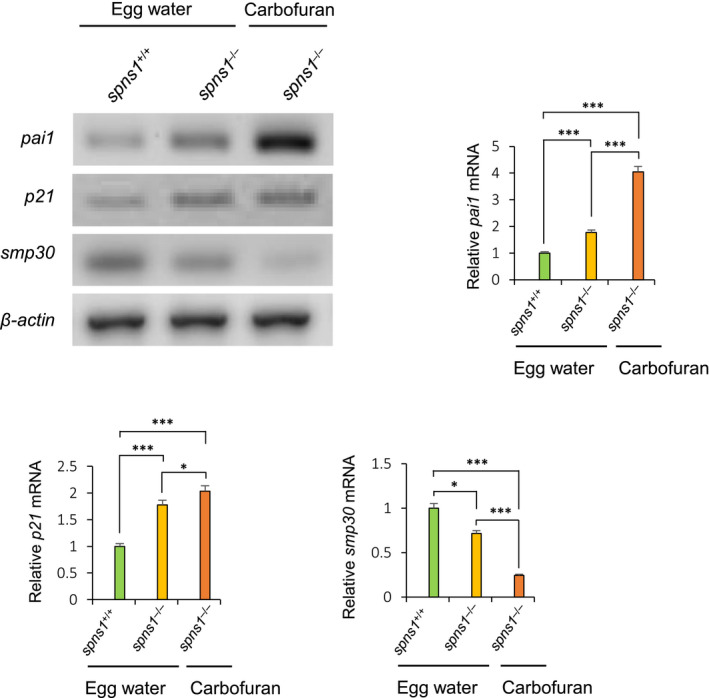
RT‐PCR analysis to determine the effect of carbofuran on *pai1*, *p21* and *smp30* mRNA levels. The expressions levels of *pai1* and *p21* were increased by the loss of the *spns1* gene. Carbofuran treatment further increased their expressions. The expression of *smp30* was significantly down‐regulated by carbofuran exposure in *spns1* mutant zebrafish. n = 5; *P ≤ .05; ***P ≤ .005

The impact of carbofuran exposure on ageing‐related genes regulations was examined by RT‐PCR analysis. Plasminogen activator inhibitor‐1 (*pai1*) is encoded in humans by the *SERPINE1* gene, whose up‐regulation is a risk factor for atherosclerosis.^38^ In elderly individuals, its level is elevated, which may lead to multiple pathologies such as vascular sclerosis, emotional stress, insulin resistance and obesity.^39^ Another gene, *p21*, is a cyclin‐dependent kinase inhibitor, promotes cellular senescence.^40^ In both prematurely aged mice and normal aged mice, the *p21* level was found elevated.^41^ In our investigation, the expression levels of both *pai1* and *p21*mRNA were significantly up‐regulated by carbofuran exposure to *spns1* mutant fish (Figure 5). On the other hand, senescence marker protein 30 (Smp30) is a calcium‐binding protein, was first recognized as a down‐regulated protein in elderly individuals.^42^ The expression of *smp30* was down‐regulated by the carbofuran treatment to *spns1* mutant fish (Figure 5).

## 4 DISCUSSION

**FIGURE 6:**
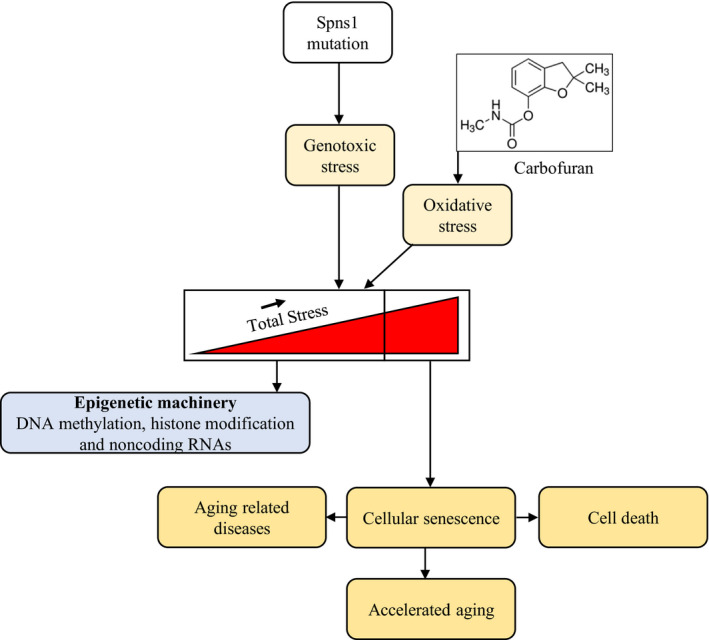
A proposed schematic model for the senescence and ageing effect of carbofuran under *spns1* defective condition. Cellular untoward events are prevented at low to medium stress conditions by epigenetic adjustment. The Spns1 defect via strong genotoxic stress induces untoward events, such as senescence, ageing, ageing‐related diseases and cell death. Carbofuran‐induced oxidative stress accelerates these untoward cellular events in *spns1* defective animals

Carbofuran is a broad‐spectrum pesticide, which is non‐specific in its action.^43^ It also exerts its toxic effects on non‐target animals, including fishes, beneficial arthropods and humans.^43^, ^44^ In mammals, carbofuran exposure leads to oxidative stress, which in turn induces neuronal injury and neuronal cell death through the inhibition of acetylcholinesterase (AChE) activity.^17^, ^27^ It was demonstrated that the inhibition of AChE by carbofuran is mediated by the carbamylation of the serine molecule of AChE.^17^ Although the toxicity of carbofuran is dose‐dependent, it can induce oxidative stress in the brain at sub‐lethal doses.^17^ The major ROS species in the body are generated via a disorganized electron transfer process of the mitochondrial respiratory chain.^46^ At a tolerable extent, ROS that generated in mitochondria may activate an adaptive defence mechanism to prevent unexpected outcome.^47^ ROS by acting as nucleophilic compounds might cause epigenetic modification of DNA (such as DNA methylation, histone modification) that control genes transcription and expression.^48^ The epigenetic machinery copes with the advanced homeostasis impairment, which reshapes the nervous, cardiovascular and respiratory systems in the elder individuals.^49^ When the ROS level exceeds the tolerable extent, the developed adaptive responses become insufficient, which leads to cellular stress.^50^, ^51^ ROS dependent cellular stress accelerates the ageing process through the induction of cellular senescence.^,^ ^47^ Oxidative stress and cellular senescence are highly linked to ageing‐related pathologies such as neurodegenerative diseases, diabetes, hypertension and atherosclerosis.^52^ A premature ageing phenotype in zebrafish is the yolk opaqueness, which was worsened by carbofuran treatment in *spns1* ^−/−^ mutant zebrafish. However, the yolk and/or yolk extension was not affected by carbofuran treatment in wild zebrafish (Figure S1). The SA β‐gal activity was weakly affected in wild zebrafish but synergistically accelerated by the carbofuran treatment in *spns1* ^−/−^ mutant fish (Figure 1C, Figure S1). Furthermore, the life span of *spns1* ^−/−^ mutant fish was shortened by carbofuran exposure. These senescence and ageing effects of carbofuran in *spns1* ^−/−^ mutant fish might be mediated by carbofuran‐induced ROS generation (Figure 6), because elevated ROS level is a key factor of ageing‐related pathologies such as neurodegenerative diseases, endocrine abnormalities and reproductive impairments.^25^, ^29^ The loss of the *spns1* gene in the mutant zebrafish induces genotoxic stress, which was revealed by yolk opaqueness, senescence and shortened life span.^14^, ^34^ Carbofuran‐induced ROS generation made additional stress in *spns1* mutant fish and exacerbated these phenotypes of the *spns1* deficiency. In the senescent cell, oxidative stress affects epigenetic machinery via DNA hypomethylation, which is considered a representative phenotype of the ageing process. In cells of an elderly individual, such as leucocytes of females of around 100 years old, age‐associated reduction of DNA methylation was manifested.^48^ In senescent cells of the *spns1* defective fish, carbofuran‐induced oxidative stress might affect the ageing process through DNA hypomethylation.

Senescence is elicited by autophagic commencement.^12^ At the beginning of autophagy, phagophore is evolved in autophagosome through the elongation of the membrane of the phagophore. During the elongation process, an ubiquitin‐like molecule LC3 is conjugated to phosphatidylethanolamine at the membrane of the autophagosome, leading to the formation of autophagosome‐associated LC3.^53^, ^54^ LC3 exists with autophagosomes until autolysosomal fusion and then delipidated and recycled.^53^ In our in vivo real‐time monitoring of EGFP‐tagged LC3 protein on *spns1* mutant context, carbofuran accelerated LC3 puncta accumulation at the same pattern as we detected the acceleration of senescence induction using SA‐β gal staining, suggesting carbofuran‐induced autophagic abnormality is associated with accelerated senescence. It was demonstrated that autophagy is usually up‐regulated by oxidative stress including cellular senescence,^8^ which is consistent with our findings.

Cellular intralysosomal waste materials are collectively known as lipofuscin, whose principal constituents are protein and lipid, and other constituents include carbohydrate, autofluorescent pigment and transition metals.^55^ The lipofuscin accumulates in several cell types (such as neurons and cardiac cells) of aged individuals. It was demonstrated that lipofuscin accumulation is associated with lysosomal dysfunction.^56^ The accumulation further increases in an ageing allied neurodegenerative disorder such as Alzheimer's disease.^56^ Under oxidative stress conditions, increased autophagosome formation leads to the delivery of undegradable oxidized protein to the lysosome that accumulates as lipofuscin.^56^ Using LysoTracker red staining and mCherry‐tagged Lamp1 expression in *spns1* deficit fish, we found excessive lysosomal expression, which was further increased by carbofuran exposure. This lysosomal overexpression upon carbofuran exposure might be because of lipofuscin accumulation under stress conditions, which need further studies to explore the mode of lipofuscinogenesis implicated with carbofuran exposure. In addition, lysosome was significantly co‐localized with autophagosome, suggesting successful autolysosomal fusion with insufficient lysosome facilitated degradation of the content of autophagosome. Such lysosomal dysfunction also validated under the *spns1* deficit stress condition.^15^ Some compounds such as chloroquine through oxidative stress and lipofuscin accumulation inhibit lysosomal proteases.^56^, ^57^ A decline in lysosomal degradation in carbofuran treated zebrafish might also be because of the inhibition of the lysosomal proteases, but it needs further studies to be confirmed. Overall, carbofuran exacerbated lysosomal dysfunction in *spns1* deficit fish.

The effects of carbofuran on developmental senescence and biological ageing are consistent with its effects on ageing allied genes regulation in *spns1* ^−/−^ mutant fish. It was demonstrated that the inhibition of the *pai1* gene down‐regulates SA‐β‐gal activity,^58^ whereas, in our investigation, SA‐β‐gal activity was concurrently increased with the *pai1* mRNA level. An increment in the *pai1* expression is associated with ageing and age‐related multimorbidity such as cognitive dysfunction, hypertension, atherosclerosis and glucose intolerance,^58^, ^59^ whereas we consistently found that carbofuran exposure shortened the life span of *spns1* mutant fish. A potent cyclin‐dependent kinase inhibitor, *p21*, whose up‐regulation is maintained in senescent cells. The intensity of senescence cells was decreased with the loss of the *p21* gene in mice.^40^ The up‐regulation of *p21* arrest cellular proliferation and promote age‐associated pathologies.^60^ It was also reported that the level of *p21* in the neuronal cells of rats was up‐regulated by carbofuran treatment.^29^ Constantly, an elevation of the *p21* mRNA level in *spns1* mutant zebrafish was observed in our investigation. On the other hand, *smp30* is a calcium homeostasis gene, protects apoptosis and prevents oxidative stress by reducing ROS generation.^60^ Carbofuran exposure to *spns1* mutant fish down‐regulated the expression of *smp30*, which might be because of additional oxidative stress in *spns1* mutant fish. Altogether, our data suggest that carbofuran accelerated cellular senescence and shortened biological ageing in *spns1* mutant zebrafish through autophagic dysregulation.

## CONFLICT OF INTEREST

The authors of this manuscript have no conflicts of interest.

## AUTHOR CONTRIBUTIONS

**Alam Khan:** Conceptualization (lead); Data curation (supporting); Methodology (lead); Writing‐review & editing (lead). **T M Fahad:** Data curation (lead); Formal analysis (lead); Investigation (lead). **Tanjima Akther:** Data curation (lead); Formal analysis (lead); Investigation (lead); Writing‐original draft (equal). **Tanjeena Zaman:** Investigation (equal); Methodology (equal); Project administration (equal). **Md Faruk Hasan:** Investigation (equal); Writing‐review & editing (equal). **Md Rafiqual Islam:** Investigation (equal). **Mohammad Saiful Islam:** Data curation (equal); Formal analysis (lead). **Shuji Kishi:** Conceptualization (lead); Writing‐review & editing (equal).

## Supporting information

Fig S1

## Data Availability

The data that support the findings of this study are available from the corresponding author upon reasonable request.
