# Supplementary figures and images for: Carbofuran accelerates the cellular senescence and declines the life span of spns1 mutant zebrafish
Source: J Cell Mol Med. 2020 Dec 4;25(2):1048–59. doi: 10.1111/jcmm.16171 (PMC7812278; doi:10.1111/jcmm.16171)

Supplementary Figure 1

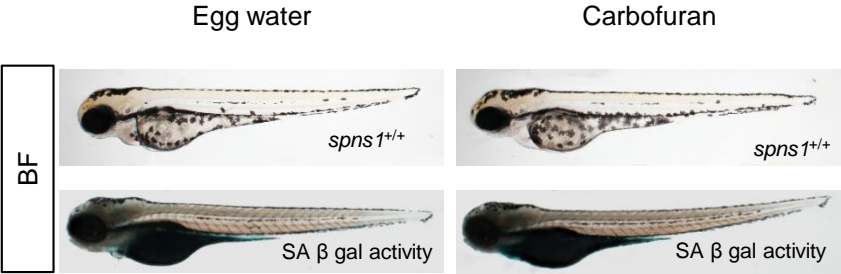

Supplement: Supplementary file 1 — Fig S1 [file JCMM-25-1048-s001.pdf]
